# Mechanistic Insight into the Pathology of Polyalanine Expansion Disorders Revealed by a Mouse Model for X Linked Hypopituitarism

**DOI:** 10.1371/journal.pgen.1003290

**Published:** 2013-03-07

**Authors:** James Hughes, Sandra Piltz, Nicholas Rogers, Dale McAninch, Lynn Rowley, Paul Thomas

**Affiliations:** 1 School of Molecular and Biomedical Science, University of Adelaide, Adelaide, South Australia, Australia; 2 Pituitary Research Unit, Murdoch Childrens Research Institute, Melbourne, Victoria, Australia; University of Michigan, United States of America

## Abstract

Polyalanine expansions in transcription factors have been associated with eight distinct congenital human diseases. It is thought that in each case the polyalanine expansion causes misfolding of the protein that abrogates protein function. Misfolded proteins form aggregates when expressed *in vitro*; however, it is less clear whether aggregation is of relevance to these diseases *in vivo*. To investigate this issue, we used targeted mutagenesis of embryonic stem (ES) cells to generate mice with a polyalanine expansion mutation in *Sox3* (*Sox3*-26ala) that is associated with X-linked Hypopituitarism (XH) in humans. By investigating both ES cells and chimeric mice, we show that endogenous polyalanine expanded SOX3 does not form protein aggregates *in vivo* but rather is present at dramatically reduced levels within the nucleus of mutant cells. Importantly, the residual mutant protein of chimeric embryos is able to rescue a block in gastrulation but is not sufficient for normal development of the hypothalamus, a region that is functionally compromised in *Sox3* null embryos and individuals with XH. Together, these data provide the first definitive example of a disease-relevant PA mutant protein that is both nuclear and functional, thereby manifesting as a partial loss-of-function allele.

## Author Summary

Alanine is one of the 20 amino acid building blocks from which proteins are generated. Nearly 500 human proteins contain stretches of consecutive alanine residues ranging from 4 to 20 amino acids in length. Whilst the function of these polyalanine (PA) tracts remains unknown, they are interesting because DNA changes (mutations) that increase their length above a threshold are responsible for nine different human disorders. *In vitro* studies indicate that expanded PA proteins misfold and aggregate, suggesting that there may be a common “gain-of-function” mechanism that underpins this group of disorders. However, these data are difficult to reconcile with genetic studies, which indicate that most PA mutations cause protein loss-of-function. Therefore, to investigate the pathological mechanism of PA disorders we generated a mouse model of X-linked Hypopituitarism (XH), a disease caused by PA expansion in the SOX3 protein. Strikingly, we found that the mouse version of the disease-causing protein was almost completely cleared from cells and that aggregates do not form *in vivo*. These data explain why this type of mutation causes protein loss-of-function and reveals why nature limits the length of PA stretches.

## Introduction

Trinucleotide repeat expansions are a relatively common cause of human disease. The expanded trinucleotide can occur in an untranslated region, for example in Fragile X syndrome in which a CGG repeat adjacent to the *FMRI* promoter causes hypermethylation and gene silencing. Alternatively repeat expansions can occur in exonic regions and result in elongation of homopolymeric amino acid tracts. For example polyglutamine (PQ) diseases such as Huntingtons disease, are associated with long unstable PQ-encoding stretches that lead to the production of a toxic species and late onset disease characterised by the loss of a subset of neurons. In addition to PQ encoding repeats, polyalanine (PA) repeat expansion has recently emerged as a significant cause of human disease. PA expansions have been linked to nine disorders of which eight are congenital and one is late onset. Each is caused by PA expansion in a separate gene, with the eight congenital disorders linked to PA expansions in developmentally-important transcription factors. These, and the associated disorders, are SOX3 (X linked Hypopituitarism), HOXA13 (hand–foot–genital syndrome), ARX (syndromic and non-syndromic X-linked mental retardation), HOXD13 (synpolydactyly type II), PHOX2B (congenital central hypoventilation syndrome), FOXL2 (blepharophimosis, ptosis and epicanthus inversus), ZIC2 (holoprosencephaly) and RUNX2 (cleidocranial dysplasia). The ninth is the ubiquitous RNA binding protein PABPN1 which is associated with the late onset disease oculopharyngeal muscular dystrophy (OPMD).

Despite considerable functional analyses, the mechanism by which PA expansion mutations cause disease is not completely understood. Phenotype/genotype correlations in humans and mouse models indicate that many PA alleles give rise to disease phenotypes that resemble (*HOXA13*, *FOXL2*, *ZIC2*) or are less severe (*ARX*) than null alleles, consistent with complete or partial loss-of-function (LOF) [1], [2], [3], [4]. In contrast, some PA alleles cause similar but more severe disease phenotypes than null alleles consistent with toxic gain-of-function (GOF) and/or dominant negative activity (*HOXD13*, *PHOX2B*) [5], [6]. Despite these differences in mode of inheritance, all PA proteins behave very similarly *in vitro*, such that over-expression in cell culture results in the generation of cytoplasmic and/or nuclear aggregates, which are likely to arise through protein misfolding [7], [8], [9]. While the relevance of cellular aggregates to PA disease in general is unclear, nuclear inclusions that contain mutant PAPBN1 protein are a hallmark of OPMD [10], suggesting that aggregates may also form in patients with PA expansion mutations in developmental transcription factors. Protein aggregation also occurs in the related polyglutamine (PQ) disorders where PQ expansion confers toxic GOF [11], [12]. Together, these data suggest that aggregate formation may have a pathogenic role in PA disease alleles with GOF activity. However, the critical question of whether aggregates form *in vivo* and, if so, how they may be implicated in the pathogenesis of LOF PA diseases remains unresolved.

To investigate these issues, we used targeted mutagenesis of ES cells to generate a 26 alanine PA tract expansion mutation in *Sox3* (*Sox3*-26ala). The analogous human mutation causes X linked Hypopituitarism (XH), a disease in which hemizygous males have GH deficiency (resulting in short stature) and fully penetrant intellectual disability [13]. Importantly, we find no evidence of SOX3-26ala aggregate formation in neural derivatives of targeted ES cells *in vivo* or in neurodifferentiated ES cell cultures. Instead, the *Sox3*-26ala mutation leads to a massive reduction in SOX3 protein within the nucleus. We also present developmental and biochemical evidence that residual mutant protein retains some activity, indicating that *Sox3*-26ala functions as a partial LOF allele.

## Results/Discussion

**Figure 1:**
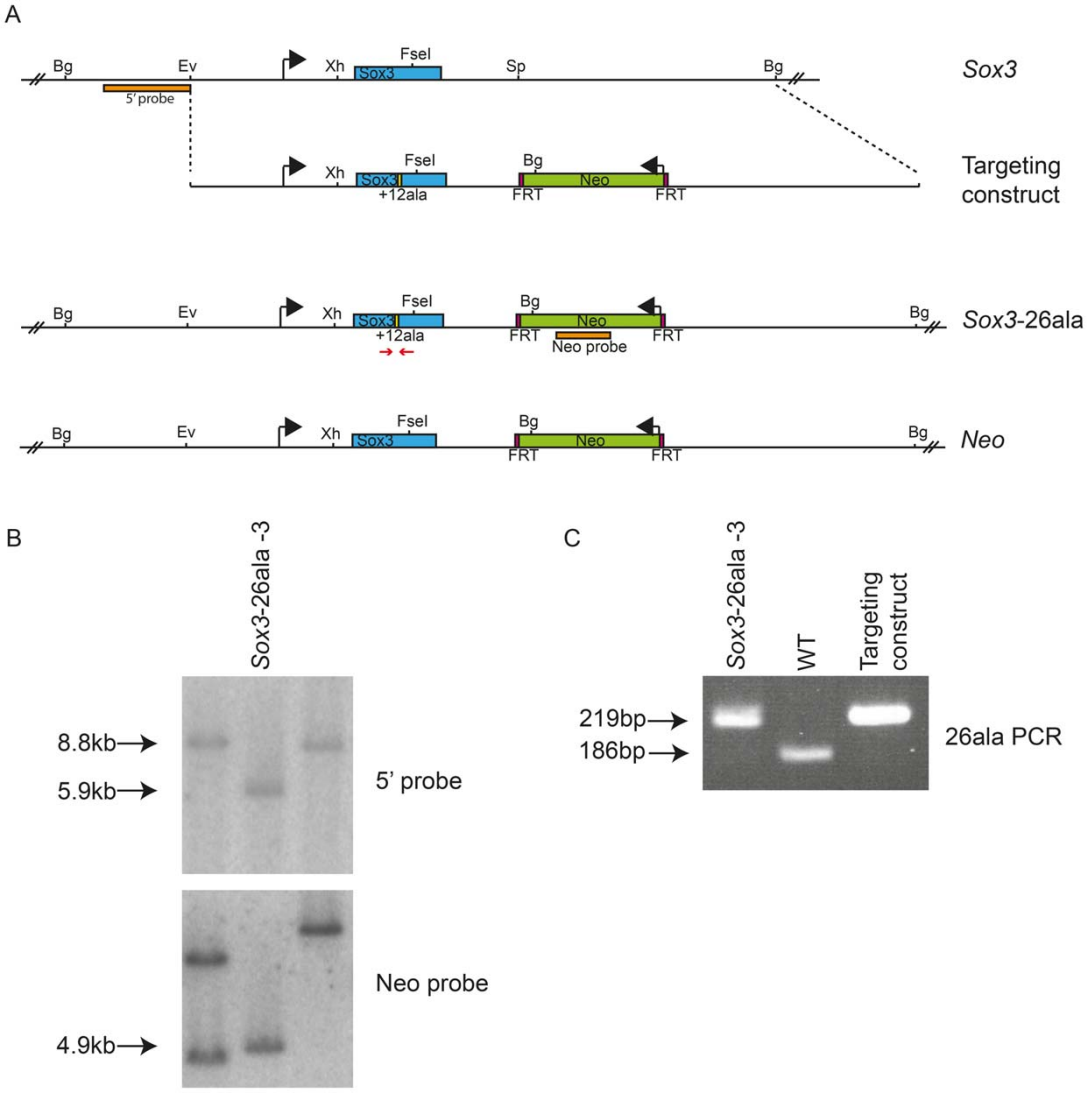
Generation of *Sox3*-26ala ES cells. Scale representation of the *Sox3* locus, targeting vector and recombinant alleles (A). Probing of *BglII* digested DNA from ES cell clones with the 5′ probe yielded an 8.8 kb fragment from the WT locus and a 5.9 kb fragment when the *Neo* cassette was recombined into the *Sox3* locus (*Sox3*-26ala or *Neo*). B) Representative Southern blot of 3 clones including a targeted clone (*Sox3*-26ala-3) is shown. C) PCR using primers spanning the alanine expansion (red arrows in A) was used to distinguish whether targeted clones carried the expansion and gave a 219 bp product instead of 186 bp as seen in WT.

In order to study the effects of disease-associated PA expansion at a cellular level we created R1 ES cells with a targeted mutation of *Sox3*. Homologous recombination was used to generate XY ES cells carrying a 36 bp expansion in the first PA tract of *Sox3*, extending the tract from 14 to 26 alanines (referred to hereafter as *Sox3*-26ala ES cells; Figure 1). Morula injection of mutant ES cells resulted in chimeras with up to 95% mutant cell contribution as assessed by coat colour (Table 1). None of these chimeras displayed any evidence of growth retardation (consistent with the absence of short stature in heterozygous female carriers of the human *SOX3*-26ala mutation who also contain a mixture of WT and mutant cells, due to X inactivation [13]). However, despite extensive breeding, none of these chimeras exhibited germline transmission of the mutant allele. In contrast, germline competence was demonstrated with WT parental ES cells as well as a clone carrying the PGK-*Neo* cassette without the PA tract expansion (referred to hereafter as *Neo* ES cells). These data suggest that the PA mutation in *Sox3* had caused a block in male fertility. This is consistent with a recently published report demonstrating that *Sox3* LOF in the germ cell lineage resulted in an early defect in spermatogenesis and the lack of any reported transmission of the human *SOX3*-26ala allele from hemizygous males [13], [14].

**Table 1:** Failure of *Sox3*-26ala targeted ES clones to transmit through the germline.

Clone	Number of chimeras	Percentage chimerism (range)	Number of pups born	Percentage of ES-derived pups (agouti)
WT	15	5–100	50	18
*Neo*	4	50–90	35	49
*Sox3*-26ala-1	6	5–50	127	0
*Sox3*-26ala-2	4	60–90	81	0
*Sox3*-26ala-3	3	20–60	53	0
*Sox3*-26ala-4	4	75–95	156	0

**Figure 2:**
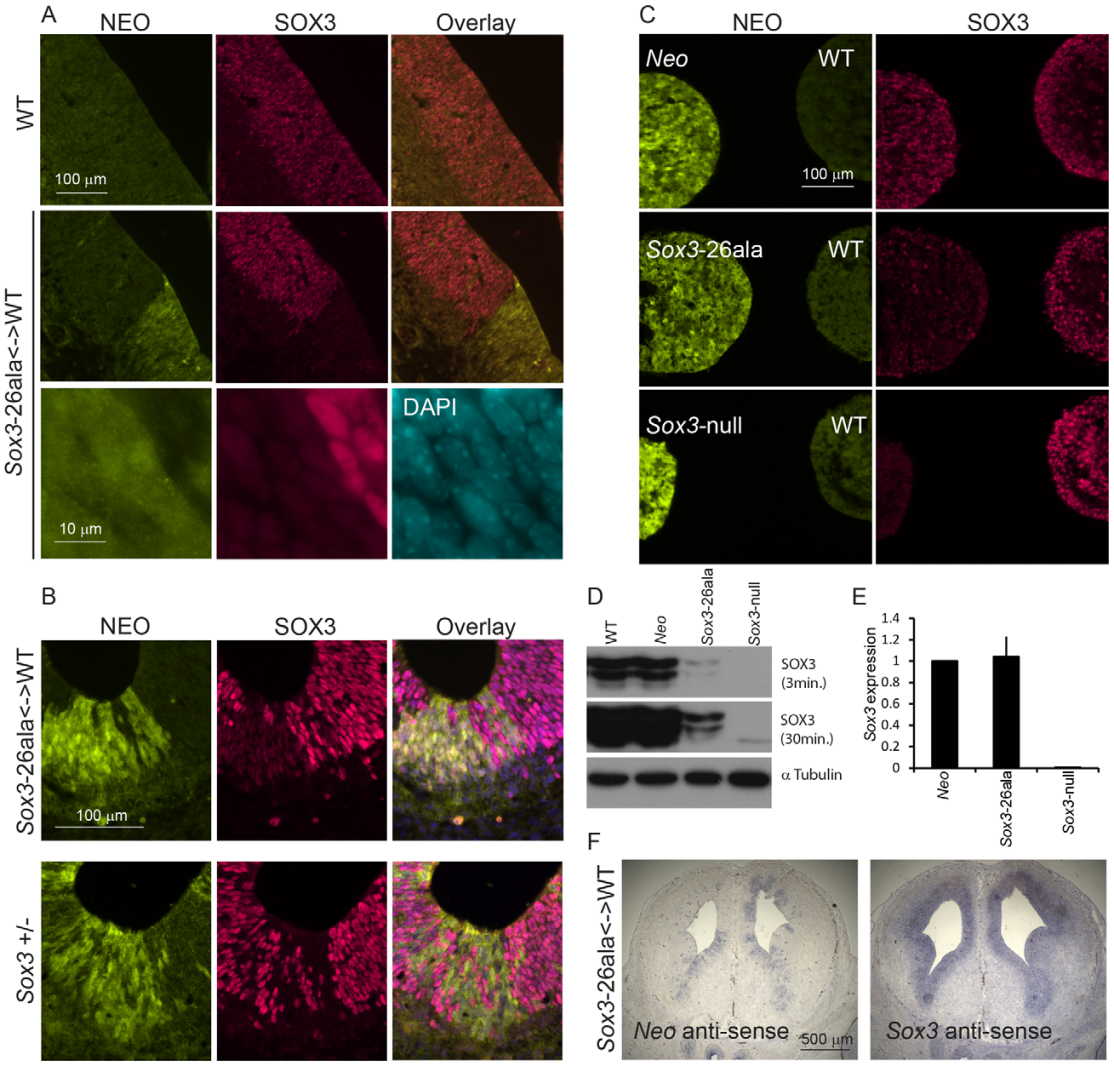
Transcription is unaffected but protein is cleared from mutant cells. A) SOX3 protein is present in every WT cell (NEO−) of the 13.5 dpc telencephalic ventricular zone but virtually absent from equivalent tissue derived from *Sox3*-26ala cells (NEO+). B) Comparison of SOX3 immunostaining on *Sox3*-null cells (from a 14.5 dpc +/− embryo) and *Sox3*-26ala expressing cells (from a *Sox3*-26ala <-> WT chimera) confirming that the antibody is SOX3-specific and that the *Sox3*-26ala expressing cells exhibit a low level of residual nuclear protein. C) WT, *Neo*, *Sox3*-26ala and *Sox3*-null ES cells were differentiated for 5 days in CDM as multi-cellular bodies. Rare SOX3 positive cells were detected in *Sox3*-26ala CDMs while the majority of cells had low SOX3 protein levels in comparison to neighbouring WT CDM bodies processed on the same slide. D–E) WT, *Neo*, *Sox3*-26ala and *Sox3*-null ES cells were grown in N2B27 for 4 days to form neural progenitors. Western blotting for SOX3 reveals a dramatic reduction of protein in *Sox3*-26ala cells (D); 3 and 30 minute exposures are shown. E) Transcript levels of *Sox3* are not affected in *Sox3*-26ala cells as determined by qPCR. Three experimental replicates are shown. Data was normalised to *Sox3* levels in*Sox3*-*Neo* control cells and error bars represent SEM. F) ISH confirms that *Sox3* transcript is present at comparable levels in ventricular zone cells at 13.5 dpc derived from both WT (*Neo*−) and *Sox3*-26ala (*Neo*+) cells. ISH performed on adjacent 10 µm coronal sections of 13.5 dpc chimeric telencephalon.

**Figure 3:**
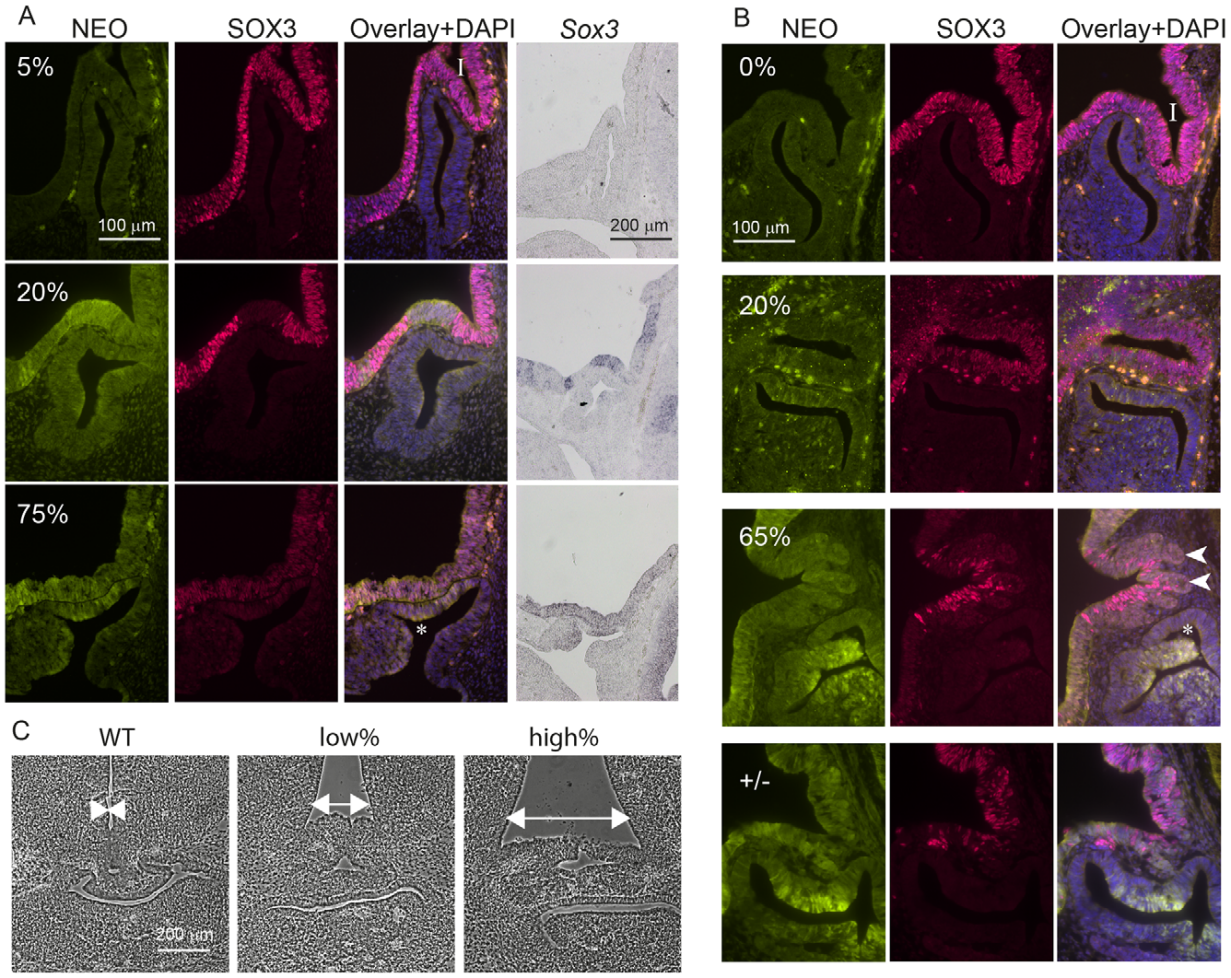
*Sox3*-26ala cells cause pituitary defects indistinguishable from *Sox3*-null cells. WT, *Sox3*+/− or *Sox3*-26ala<->WT chimeras were cut sagittally at 11.5 dpc (A) or 13.5 dpc (B) and immunostained for SOX3 and NEO expression. Percentage chimerism for each embryo in (A) and (B) was determined by qPCR as outlined in the methods. ISH for *Neo* on adjacent sections at 11.5 dpc confirmed the identification of mutant cells within the ventral diencephalon (A). Examples at 11.5 dpc show the infundibulum (I) appears unaffected in a 5% chimera, shallow in a 20% chimera and absent in a 75% chimera that also displayed a Rathke's Pouch (*) that had failed to detach from the oral ectoderm. At 13.5 dpc, heterozygous and high percentage (65%) chimeric embryos displayed a distorted infundibulum (I) with a lobular edge (arrow heads) and a branched Rathke's Pouch (*). Low percentage chimeras (20%) look similar to WT (0%). C) Phase micrographs of 13.5 dpc coronal sections through the developing pituitary show a broadening at the base of the third ventricle in chimeras (arrows). Chimerism for embryos shown in (C) was determined based on immunoreactivity for NEO in adjacent sections (data not shown).

Since we were unable to transmit the *Sox3*-26ala mutation through the male germline, the phenotypic consequences of the mutation were initially investigated in *Sox3*-26ala <-> WT chimeric embryos. Immunostaining with a SOX3-specific antibody revealed a dramatic reduction in SOX3 protein in 13.5 dpc telencephalic ventricular zone cells expressing the *Sox3*-26ala allele (identified by NEO-immunoreactivity) in comparison to neighbouring WT cells (Figure 2A). The ability of the antibody to detect mutant protein was confirmed by staining COS-7 cells expressing exogenous mouse *Sox3*-26ala, in which large peri-nuclear and cytoplasmic aggregates were common (Figure S1). A striking reduction in mutant SOX3 protein was also observed in 7.5 dpc, 9.5 dpc, 10.5 dpc and 11.5 dpc chimeras (Figure 3 and Figure S2) indicating that this phenotype was not stage-dependent. High power microscopy failed to detect any cytoplasmic or nuclear aggregates of mutant protein but did reveal a very low level of protein in the nucleus (Figure 2A). Comparison of *Sox3*-26ala and *Sox3*-null embryonic CNS cells confirmed that the low level of SOX3-26ala protein that we observed was not due to background signal (Figure 2B). Mutant cells appeared morphologically normal and there was no apoptotic induction as assessed by staining for activated Caspase3 (Figure S3). To further characterise the cellular phenotype of *Sox3*-26ala mutant cells, we performed neurodifferentiation of *Sox3*-26ala ES cells (which are XY and therefore lack a WT *Sox3* allele). Immunohistochemical analysis confirmed the overwhelming reduction of nuclear SOX3 protein in mutant cultures (Figure 2C) in which the expression of other neural progenitor markers were unaffected (data not shown). Rare cells with near normal levels of nuclear mutant protein were also detected but in no case was there evidence of protein aggregation (Figure 2C). Western blot analysis further supported the near complete loss of steady-state SOX3 protein levels in mutant cells (Figure 2D). As triplet repeat expansion mutations have been shown to affect mRNA transcription [12], we compared *Sox3* message levels in WT and mutant cells *in vitro* and *in vivo* using qPCR and *in situ* hybridisation, respectively (Figure 2E–2F). No difference in *Sox3* mRNA level was detected, indicating that the massive reduction in SOX3 protein levels in the mutant cells has a post-transcriptional aetiology and presumably occurs via degradation of misfolded protein [8]. This was supported by *in vitro* transcription/translation analysis which indicated that translation of the mutant transcript was unaffected by the PA mutation (Figure S4).

Taken together, these results indicate that *Sox3*-26ala is a LOF allele and argue strongly against a role for aggregation in the pathogenesis of XH. To further investigate LOF as the mechanism of disease, we compared the development of the hypothalamus, infundibulum and anterior pituitary in *Sox3*-26ala chimeric embryos to *Sox3* heterozygous embryos that carry a complete LOF (null) allele. *Sox3* +/− embryos exhibit a defect in infundibular development that results in aberrant induction and bifurcation of the anterior pituitary primordium, Rathke's Pouch (RP) [15] accompanied by expansion of the floor of the ventral diencephalon (Figure 3). Similar defects in infundibular morphology and pituitary localisation have also been identified in XH patients [16]. Importantly, 13.5 dpc and 11.5 dpc high percentage *Sox3*-26ala <-> WT chimeras exhibited dysmorphology of the infundibulum, Rathke's Pouch and ventral diencephalon that was indistinguishable from *Sox3*+/− embryos (Figure 3). These abnormalities were also present in low percentage chimeras, although were less severe consistent with the loss of SOX3 function being responsible for this phenotype. Notably, there was an obvious reduction in SOX3 protein in mutant (NEO+) cells thereby confirming that PA expansion has a functional impact in neural cells that are directly implicated in XH pathology.

**Figure 4:**
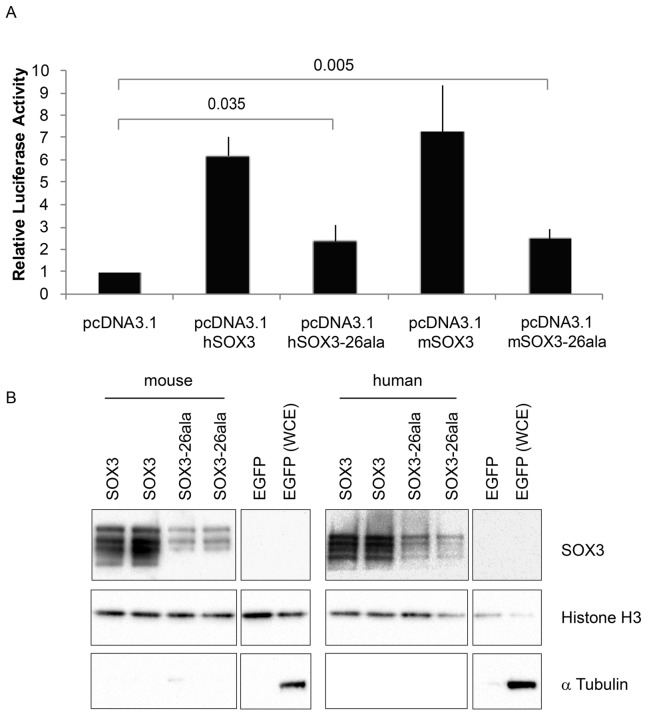
SOX3-26ala from mouse and human retains transactivation activity. A) COS-7 cells were transfected with pcDNA3.1 expression vector containing either mouse *Sox3*, human *SOX3*, mouse *Sox3*-26ala, human *SOX3*-26ala or an empty vector control. Values represent mean normalised luciferase values plus standard deviation of four independent experiments measured 48 hours after transfection. Student's T-tests (two tailed, unequal variance) of SOX3-26ala from human or mouse compared to empty vector control show a statistically significant increase in luciferase activity. B) Nuclear protein lysates prepared from duplicate plates 48 hours after transfection show that less SOX3 is detected in the nucleus of cells expressing both mouse and human SOX3-26ala. pcDNA3.1-EGFP transfected cells were used as a control and prepared for both nuclear protein and whole cell extracts (WCE). Blotting for Histone H3, indicates equal loading and blotting for α-Tubulin shows an absence of cytoplasmic contamination in nuclear preparations. Transfection efficiency was determined by co-transfecting EGFP and counting positive cells prior to harvesting and found to be equal for all plasmids.

**Figure 5:**
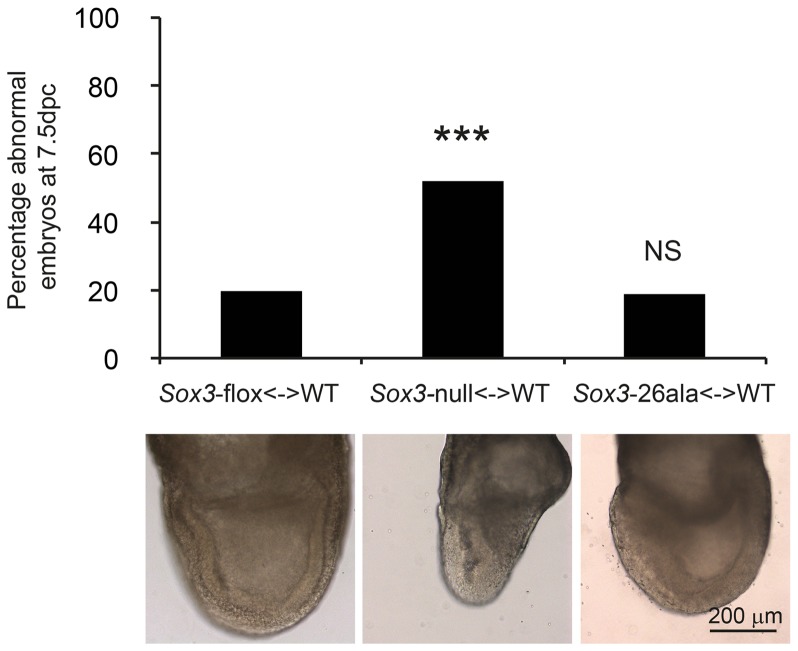
Residual nuclear SOX3-26ala protein rescues a gastrulation defect of *Sox3*-null <-> WT chimeric embryos. *Sox3*-26ala <-> WT chimeras are normal at 7.5 dpc (gastrulation) unlike *Sox3*-null <-> WT chimeras. A total of 15 *Sox3*-flox<-> WT ES chimeras, 31 *Sox3*-null<-> WT chimeras and 21 *Sox3*-26ala<-> WT chimeras were blind scored by two independent operators as morphologically normal or abnormal. The average score for each embryo was used to plot the percentage of abnormal embryos for each condition and chi squared analysis was performed with *Sox3*-flox<-> WT embryos used to set expected outcomes. Significantly more *Sox3*-null<-> WT chimeras were abnormal (p = 0.0001) while *Sox3*-26ala<-> WT chimeras did not deviate from expected (p = 0.95). An example of normal morphology is shown for *Sox3-*flox<-> WT and *Sox3*-26ala<-> WT chimeras and an abnormal *Sox3*-null<-> WT chimera is also shown that exhibits distortion of the ectodermal layer and apparent expansion of cells at the primitive streak and the adjacent extra-embryonic region.

Having established that *Sox3*-26ala behaves as a LOF allele, we next considered whether the low level of remnant SOX3 protein in mutant cells was functional. To investigate this, we initially performed transactivation assays in COS-7 cells using wild type and mutant human and mouse SOX3 expression constructs and a luciferase reporter containing four SOX consensus motif (SOCM) binding sites. Both mouse and human SOX3-26ala proteins showed activity in this assay that was significantly higher than background (Figure 4A). However, consistent with previous reports [7], [16], this activity was much lower than WT SOX3 protein. To investigate whether this reduction was caused by lower nuclear protein levels or an inherent defect in mutant protein transactivation activity, we measured that relative amount of WT and mutant SOX3 protein in the nucleus of transfected cells by Western Blot (Figure 4B). We observed a reduction of similar magnitude to the reduced luciferase output, suggesting that the mutant protein that is present in the nucleus has similar activity to WT. To investigate whether the residual nuclear SOX3-26ala protein is functional *in vivo* we compared the phenotypes of 7.5 dpc chimeras generated from either *Sox3*-null or *Sox3*-26ala ES cells, as it has been reported that *Sox3*-null ES cell <-> WT embryo chimeras exhibit severe gastrulation defects [15]. As previous analysis of *Sox3*-null ES cell chimeras was not performed with R1 ES cells (the parent line used to generate the 26ala mutant ES cells), we generated R1 *Sox3*-null ES cells for this experiment. Consistent with previous reports, a high proportion (52%) of *Sox3*-null ES cell chimeras generated abnormal gastrulae (Figure 5). In contrast, only 19% of *Sox3*-26ala ES cell <-> WT embryo 7.5 dpc chimeras were morphologically abnormal which was not significantly different to the proportion of abnormal chimeric embryos generated using *Sox3*-flox control ES cells (Figure 5). Immunostaining of *Sox3*-26ala chimeras revealed a reduction in SOX3 protein levels that was similar to later embryonic stages (Figure S1). These data indicate that nuclear SOX3-26ala protein is functional *in vivo* and is sufficiently abundant to prevent overt defects in gastrulation. We therefore conclude that *Sox3*-26ala is a partial LOF allele.

This study represents the first investigation of the disease-associated *Sox3*-26ala mutation under the control of the endogenous locus in a whole animal setting. The complete lack of aggregates in SOX3 positive CNS zones (including the hypothalamic-pituitary axis) provides strong evidence that aggregation is not a feature of the human disease. In contrast, our data demonstrate that PA expansion results in a dramatic reduction in SOX3 protein due to a post-translational defect. Given that PA expansion proteins co-localise with chaperones *in vitro* and that aggregation is promoted by pharmacological inhibition of the proteasome [8], it seems likely that mutant SOX3 protein misfolds and is cleared from the cell. Of note, the small amount of protein that remains translocates to the nucleus and is functionally active. The level of protein that remains is sufficient for some developmental contexts but not others (i.e. gastrulation but not pituitary induction). This context-specific threshold of SOX3 activity may explain why XH patients with *SOX3* PA mutations, but not null mutations, have been identified.

In direct contrast to the *Hoxd13* (+7ala) *spdh* spontaneous mouse model in which mutant protein is mislocalised to the cytoplasm [8], our data provide the first definitive example of a disease-relevant PA mutant protein that is both nuclear and functional, thereby manifesting as a partial LOF allele. Interestingly, mutant protein levels in the *Hoxa13* and *Arx* PA mouse models, which also exhibit LOF inheritance, are diminished but not abolished by western blot and whole mount immunodetection [1], [17]. This raises the possibility that nuclear localisation of suboptimal levels of functional protein may be a feature of several PA diseases. In the case of ARX, the PA mutant mouse model has been reported to have normal nuclear localisation of mutant protein in some tissues, but to have a reduction in the total number of positive cells [17], [18]. Given our data, an alternative interpretation is that cells expressing ARX are not lost but are unable to be detected due to misfolding and clearance of the mutant protein. In situ hybridisation of *Arx* transcripts should provide a means of discriminating between these possibilities.

In addition to *SOX3*-26ala, XH is also associated with a 7 alanine expansion mutation (*SOX3*-22ala) in the same polyalanine tract [16], [19], [20]. Although *SOX3*-22ala is occasionally associated with mild learning difficulties [20], it is interesting to note that the infantile behaviour/severe MR that is fully penetrant in *SOX3*-26ala-carrying males is not found in affected males with the *SOX3*-22ala expansion mutation, suggesting that the shorter expansion is less severe. Given our data showing a massive depletion of SOX3 protein in *Sox3*-26ala mutant cells *in vivo*, we would predict that SOX3-22ala protein would also be reduced (compared to WT) but to a lesser extent than the SOX3-26ala protein. This higher level (and therefore activity) of SOX3-22ala compared to SOX3-26ala would be sufficient for “cognitive” CNS development. Consistent with this idea, the SOX3-22ala protein is less prone to aggregate formation in cell culture [7], [16], although one must be cautious in interpreting pathological mechanism from over expression studies in heterologous cell lines. While it would be useful to directly compare SOX3-26ala and SOX3-22ala levels *in vivo*, a mouse model of the *SOX3*-22ala mutation has not been generated. However, given that it is now possible to generate neural precursors directly from patients via iPS cells, it would be interesting to compare the SOX3 protein levels in neuroprogenitors derived from *SOX3*-22ala and *SOX3*-26ala carrying males. This approach might also be informative for PA disorders in general, particularly given that increased disease severity is generally associated with longer PA expansions [21].

Based on these and other published data, we propose that both GOF and LOF PA disease alleles are associated with a primary defect in protein folding but that a critical difference in the capacity of cells to clear mutant protein results in either the accumulation of mislocalised cytoplasmic protein (GOF) or a diminished level of functional nuclear protein (LOF). As indicated by earlier reports [21], the former mechanism provides scope for dominant-negative and toxic GOF through cytoplasmic sequestration of endogenous binding partners. In the latter, we propose that misfolded protein is efficiently cleared by the cell without significant perturbation of other cellular processes, manifesting as LOF or partial LOF, depending on the amount of residual functional protein. Factors that determine whether or not a cell is able to efficiently clear the misfolded protein could include the level of expression, local concentration differences within the cell, the efficiency of the degradation pathway within different affected cell types and the length of the PA expansion. We propose that the aggregates seen when SOX3-26ala and all other PA expansion proteins are expressed *in vitro* reflect the overloading of the cell with mutant protein such that normal degradation pathways are overwhelmed. Resolution of the factors that determine whether a cell is able to clear or tolerate mutant protein will have broader implications for proteinopathies such as Alzheimers Disease and Huntingtons Disease in which only subsets of cells display aggregation. For the future, it will be interesting to determine the behaviour of mutant protein in other PA mouse models with GOF and LOF inheritance and, where possible, in patient-derived induced pluripotent stem (iPS) cell derivatives.

## Methods

### ES cell targeting

#### *Sox3*-26ala allele

The targeting vector was based on a *Sox3-Neo* floxed vector published previously in which GFP and the loxP sites were removed and a 36 bp insertion was introduced to the existing 42 bp alanine encoding tract [15]. This was electroporated into 129 strain-derived (R1) mouse ES cells and 1000 G418 resistant clones were screened by Southern blotting. Integration at the *Sox3* locus was determined by a shift in a *BglII* fragment from 8.8 kb to 5.9 kb when probed with a 5′*Sox3* probe [15]. Clones were subsequently screened for the alanine expansion using PCR primers flanking the alanine tract; 5′-AGACGCTGCTCAAGAAGGAC-3′ and 5′-CTGCACGAGCGAGTAGGC-3′. Clones targeted with the *Neo* cassette but lacking the *Sox3*-26ala expansion were designated (*Neo*).

#### Null allele

The targeting vector for generation of the null allele was the *Sox3*-*Neo* floxed vector published previously [15]. 400 clones were screened using the above probe and homologous recombinants identified by a shift in the *BglII* fragment from 8.8 kb to 7.5 kb. Two correctly targeted clones were transiently transfected with a Cre recombinase expression plasmid (kind gift from Duncan Hewett), seeded at clonal density and screened by PCR for the absence of *Sox3*. Putative *Sox3* null clones were confirmed by Southern blotting (5 kb *BglII* fragment using the 5′*Sox3* probe, as above).

### Chimera generation

129 derived ES clones were injected into c57/Bl6xB6D2F1 2.5 dpc morula, cultured overnight and transferred as blastocysts into the uterus of 2.5 dpc pseudopregnant recipients.

### ES cell culture and neurodifferentiation

R1 ES cells were maintained on irradiated MEFs in standard conditions and neurodifferentiated either in aggregates in chemically defined media as described in [22] referred to as (CDM) or as monolayers as previously described [23], referred to as (N2B27).

### qPCR gene expression and estimation of percentage chimerism

RNA was prepared with Trizol (Invitrogen) and reverse transcribed with a High Capacity RNA-to-cDNA kit (ABI). qPCR was performed using Fast SYBR Green Master Mix (ABI) and run on an ABI 7500 StepOnePlus system. Primer sequences and lengths of amplified products were: *Sox3* (117 bp) 5′-GAACGCATCAGGTGAGAGAAG-3′ and 5′-GTCGGAGTGGTGCTCAGG-3′, β*-Actin* (89 bp) 5′-CTGCCTGACGGCCAGG-3′ and 5′-GATTCCATACCCAAGAAGGAAGG-3′. *Sox3* expression was normalised to β*-Actin* and expressed as relative quantity (RQ) using ABI software. Embryonic chimerism was determined against a standard curve of *Sox3* dosage generated from adult gDNA of WT and *Sox3*-null mixed 0∶100, 25∶75, 50∶50, 100∶0. Loading was corrected using primers against *Sox1* (171 bp) 5′-GACTTGCAGGCTATGTACAACATC-3′ and 5′-CCTCTCAGACGGTGGAGTTATATT-3′ and *Ngn3* (120 bp) 5′-CCCCAGAGACACAACAACCT-3′ and 5′-AGTCACCCACTTCTGCTTCG-3′. Chimerism in Figure 2C was estimated by NEO IF and ISH.

### ISH and IF

Embryos were fixed in 4% PFA overnight and CDM bodies were fixed for 15 minutes. Both were then equilibrated in 30% sucrose overnight, set in OCT compound and cryosectioned on a Leica CM1900 at 10 µm. ISH on fixed frozen sections was performed using a *Sox3* probe from a fragment generated with the following primers; 5′-AGCGCCTGGACACGTACAC-3′ and 5′-AGCGCCTGGACACGTACAC-3′ and a *Neo* probe from a fragment amplified with the following primers; 5′-GATCGATCCCCTCAGAAGAAC-3′ and 5′-GGCTATTCGGCTATGACTGG-3′. Images were captured on a Zeiss Axiophot upright microscope with AnalySIS software, using a 2.5× (Zeiss Neofluar; NA0.5) or a 10× (Zeiss Neofluar; NA0.3) objective lens. Primary antibodies and dilutions were goat anti-hSOX3 (R&D #AF2569,1∶150) and rabbit anti-NTPII (Millipore #06-747, 1∶150). Imaging of IF was performed on a Zeiss Axioplan2 upright microscope with Axiovision software, using a 20× (Zeiss Apochromat; NA0.75) or a 100× (Zeiss Apochromat; NA1.3) objective lens.

### Western blotting

Day4 N2B27 neurodifferentiated ES cells were lysed in RIPA buffer supplemented with protease inhibitors. SDS loading buffer was added and the samples were resolved by 10% SDS-PAGE. Duplicate gels were subjected to immunoblot analysis using anti-hSOX3 (1∶2500) and anti-α-Tubulin (Sigma #T8203, 1∶2500) antibodies since both detect proteins at ∼40 kD. Signals were developed using ECL substrate (West Pico, Pierce). For nuclear protein lysates, cells were subjected to hypotonic lysis (10 mM HEPES pH 7.9, 1.5 mM MgCl2, 10 mM KCl, 0.4% NP-40, 10% Ficoll-400, 1 mM DTT, 1 mM PMSF, 1× protease inhibitors), centrifugation to pellet nuclei that were then washed in wash buffer (10 mM HEPES pH 7.9, 1.5 mM MgCl2, 150 mM KCl, 10% Ficoll-400, 1 mM DTT, 1 mM PMSF, 1× protease inhibitors) and lysed in nuclear extract buffer (20 mM HEPES pH 7.9, 1.5 mM MgCl2, 0.5 mM EDTA, 20% glycerol, 0.42 M KCl, 1 mM DTT, 1 mM PMSF, 1× protease inhibitors).

### Luciferase transcription assay

Transcriptional activities of mouse and human SOX3 and SOX3-26ala were determined using the Dual-Luciferase Reporter Assay System (Promega). 1.0 µg of plasmid DNA was transfected into COS-7 cells using Lipofectamine according to manufacturer's instructions. All transfections were performed in triplicate and contained luciferase reporter Sox Consensus Motif (SOCM; 4×AACAAAG) [7], *Renilla* luciferase plasmid pRL-CMV and one of pcDNA3.1, pcDNA3.1 hSOX3, pcDNA3.1 hSOX3-26ala, pcDNA3.1 mSOX3 or pcDNA3.1 mSOX3-26ala expression constructs (Promega). The firefly luciferase and *Renilla* luciferase activities were determined after 48 h on a FluorStar Optima (BMG technologies). Relative luciferase activity is the ratio of firefly to *Renilla* normalised to pcDNA3.1. The assay was repeated four times. Statistical analysis was performed using Student's T-test (two tailed, unequal variance).

### Ethics statement

Animal experiments were approved by the University of Adelaide Animal Ethics Committee. All studies were conducted in accordance with the principles of animal replacement and reduction and experimental refinement. Animals were monitored daily for evidence of illness and, if distressed, were culled immediately by cervical dislocation by an experienced investigator/animal technician.

### Supplementary methods

#### Cell free transcription/translation

Cell free transcription/translation was performed using the TnT Coupled Reticulocyte Lysate System (Promega, #L4610) as per manufacturer's instructions. Plasmids used were 250 ng of either pcDNA3.1 hSOX3, pcDNA3.1 hSOX3-26ala, pcDNA3.1 mSOX3 or pcDNA3.1 mSOX3-26ala. Reactions were resolved on a Bio-Rad 10% precast gel (#456-1033), transferred to PVDF membrane, exposed to phosphor-screen and scanned.

#### COS-7 aggregation assay

COS-7 cells were grown on glass coverslips, transfected with one of pcDNA3.1, pcDNA3.1 hSOX3, pcDNA3.1 hSOX3-26ala, pcDNA3.1 mSOX3 or pcDNA3.1 mSOX3-26ala using Fugene6 (Roche) and stained with SOX3 antibody and DAPI two days later. At least 100 cells were scored randomly from each condition. Results are shown as the mean of three independent experiments and error bars represent one standard deviation.

## Supporting Information

Figure S1 Mutant SOX3-26ala protein aggregates *in vitro*. COS cells were transfected with mouse or human pcDNA3.1 SOX3 or pcDNA3.1 SOX3-26ala expression plasmids and two days later cells were fixed and stained with a SOX3 antibody (Green) and DAPI (Blue). Cells were scored according to the localisation of the SOX3 staining as nuclear, cytoplasmic or peri-nuclear for which representative examples are shown. At least 100 cells were scored from each condition and the experiment was repeated three times. Error bars show one standard deviation from the mean. Student's T-tests between WT and mutant for each category for both mouse and human revealed statistically significant differences with a P value of less than 0.05 in each case.
(TIF)

Figure S2 SOX3-26ala protein is cleared from neuroprogenitor cells *in vivo*. *Sox3*-26ala<->WT chimeras were cut at 7.5 dpc (transverse section through neural groove), 9.5 dpc (transverse section through neural tube) or 10.5 dpc (sagittal section through ventral diencephalon) and stained with antibodies against SOX3 and NEO. Mutant *Sox3*-26ala cells (NEO positive cells; arrow heads) have low SOX3 staining. Confocal microscopy was performed for 7.5 dpc and 9.5 dpc embryos on a Leica SP5 spectral scanning confocal microscope with AnalySIS software and a 100× (Leica HCL PL-APO; NA1.4) objective. 10.5 dpc embryos were imaged using a Zeiss Axioplan 2 epifluorescence microscopy as described in the methods.
(TIF)

Figure S3 SOX3-26ala expressing cells show no signs of apoptosis. Coronal section of a 13.5 dpc *Sox3*-26ala<->WT chimera stained with antibodies against SOX3 and Activated Caspase3 (BD Pharmingen, #559565, 1∶1000). Boxed area in overlay shows a rare Activated Caspase3 positive cell, while the arrowhead indicates a patch of mutant cells (low SOX3) in which no Activated Caspase3 positive cells are seen.
(TIF)

Figure S4 A) Phospor image of an in vitro transcription/translation reaction of mouse and human SOX3 WT and 26ala expression constructs. SOX3 protein of the expected size is indicated by the arrow. Two strong bands were generated by the mouse plasmids, possibly reflecting the inclusion of an upstream ATG in the plasmid, while human plasmids generated only one strong band at the expected size. SOX3-26ala for both mouse and human runs slightly higher than WT protein reflecting the expanded polyalanine tract. In addition, both mouse and human SOX3-26ala have a high molecular weight species that appears to be an aggregated form of SOX3 (*). (-) indicates no template control. B) Immunoblotting using anti-SOX3 antibody (R&D #AF2569) generates a near identical pattern to the phosphor image in (A), thus confirming that the antibody is able to detect mutant and wild type protein with comparable efficiency.
(TIF)
