# Supplementary figures and images for: Mechanistic Insight into the Pathology of Polyalanine Expansion Disorders Revealed by a Mouse Model for X Linked Hypopituitarism
Source: PLoS Genet. 2013 Mar 7;9(3):e1003290. doi: 10.1371/journal.pgen.1003290 (PMC3591313; doi:10.1371/journal.pgen.1003290)

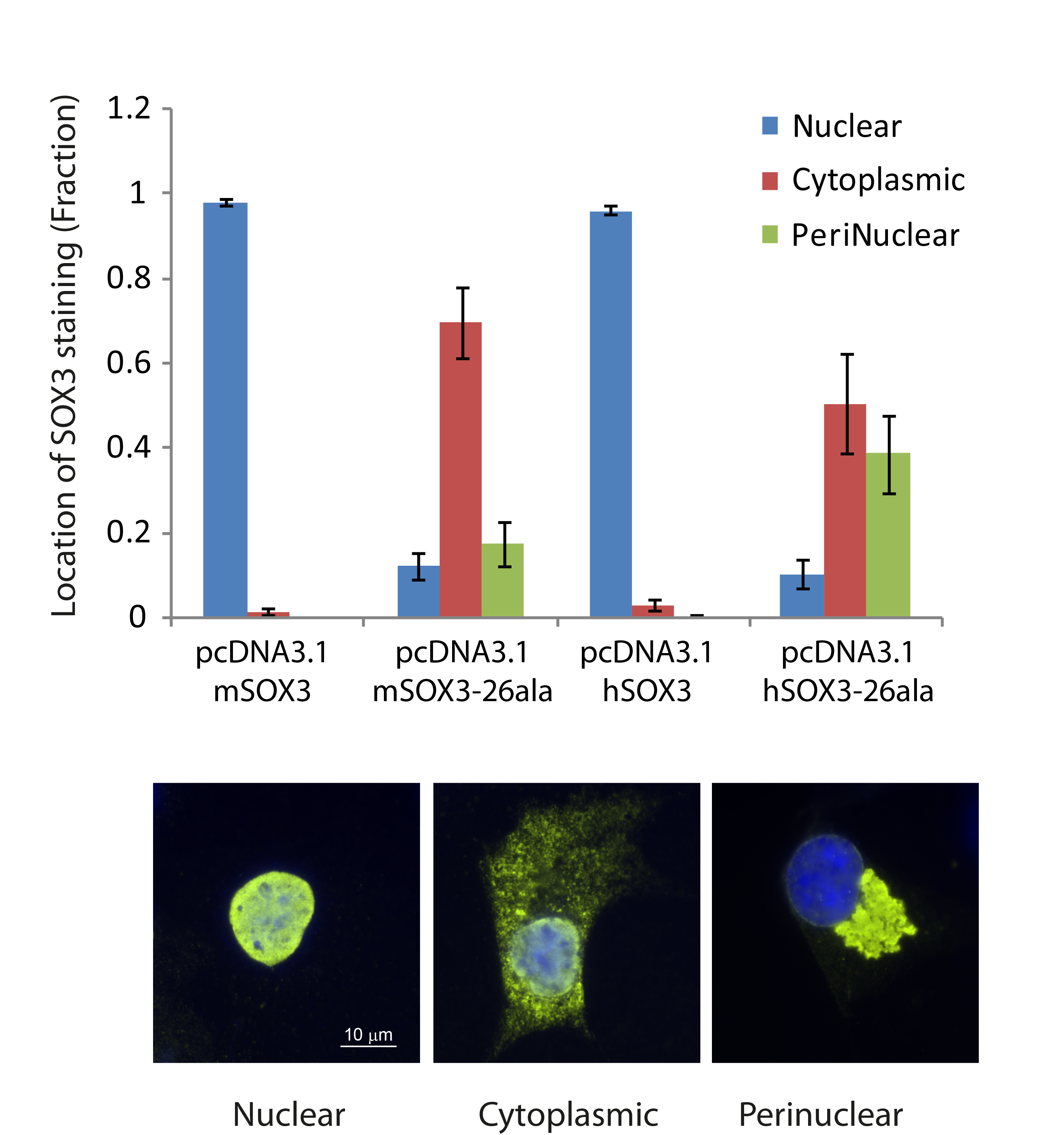

Supplement: Figure S1 — Mutant SOX3-26ala protein aggregates in vitro. COS cells were transfected with mouse or human pcDNA3.1 SOX3 or pcDNA3.1 SOX3-26ala expression plasmids and two days later cells were fixed and stained with a SOX3 antibody (Green) and DAPI (Blue). Cells were scored according to the localisation of the SOX3 staining as nuclear, cytoplasmic or peri-nuclear for which representative examples are shown. At least 100 cells were scored from each condition and the experiment was repeated three times. Error bars show one standard deviation from the mean. Student's T-tests between WT and mutant for each category for both mouse and human revealed statistically significant differences with a P value of less than 0.05 in each case. (TIF) [file pgen.1003290.s001.tif]

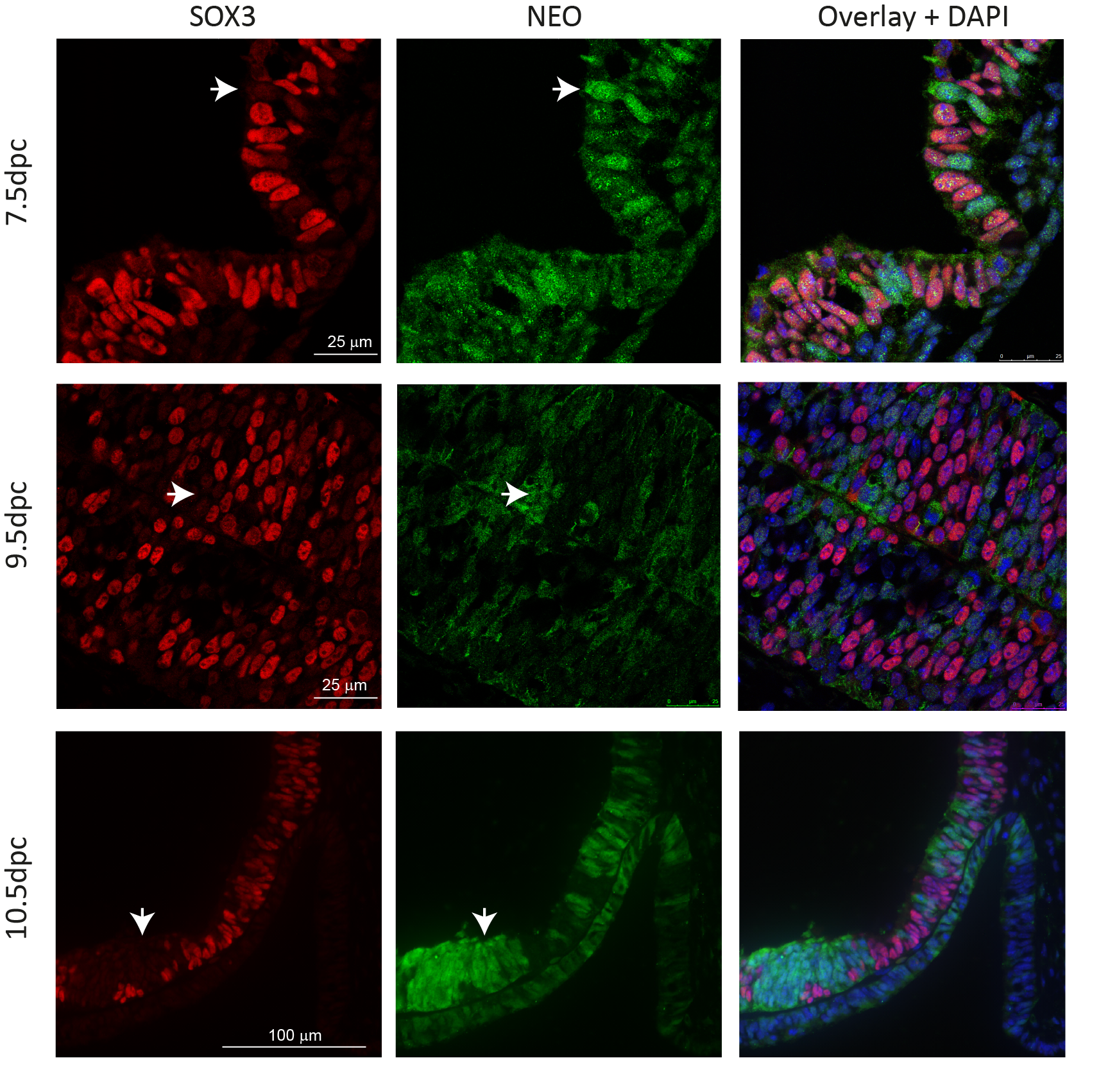

Supplement: Figure S2 — SOX3-26ala protein is cleared from neuroprogenitor cells in vivo. Sox3-26ala<->WT chimeras were cut at 7.5 dpc (transverse section through neural groove), 9.5 dpc (transverse section through neural tube) or 10.5 dpc (sagittal section through ventral diencephalon) and stained with antibodies against SOX3 and NEO. Mutant Sox3-26ala cells (NEO positive cells; arrow heads) have low SOX3 staining. Confocal microscopy was performed for 7.5 dpc and 9.5 dpc embryos on a Leica SP5 spectral scanning confocal microscope with AnalySIS software and a 100× (Leica HCL PL-APO; NA1.4) objective. 10.5 dpc embryos were imaged using a Zeiss Axioplan 2 epifluorescence microscopy as described in the methods. (TIF) [file pgen.1003290.s002.tif]

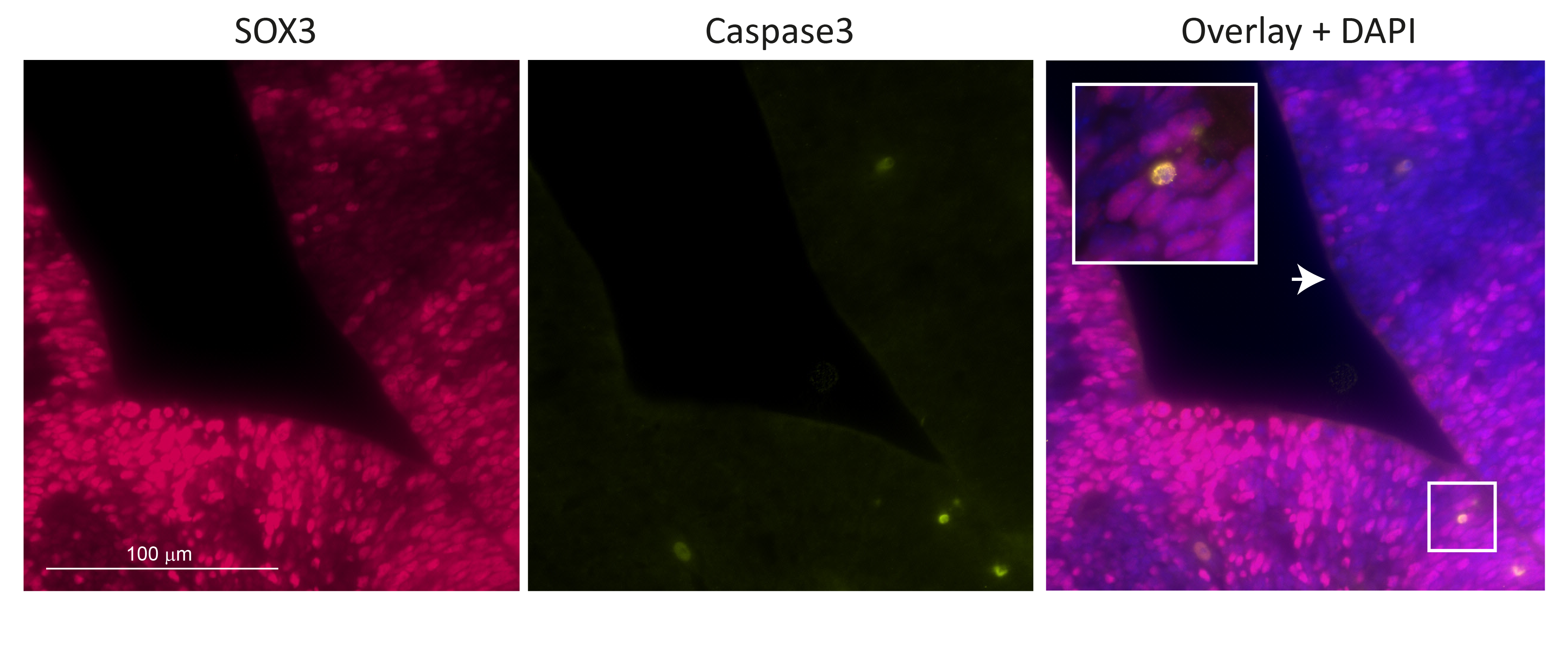

Supplement: Figure S3 — SOX3-26ala expressing cells show no signs of apoptosis. Coronal section of a 13.5 dpc Sox3-26ala<->WT chimera stained with antibodies against SOX3 and Activated Caspase3 (BD Pharmingen, #559565, 1∶1000). Boxed area in overlay shows a rare Activated Caspase3 positive cell, while the arrowhead indicates a patch of mutant cells (low SOX3) in which no Activated Caspase3 positive cells are seen. (TIF) [file pgen.1003290.s003.tif]

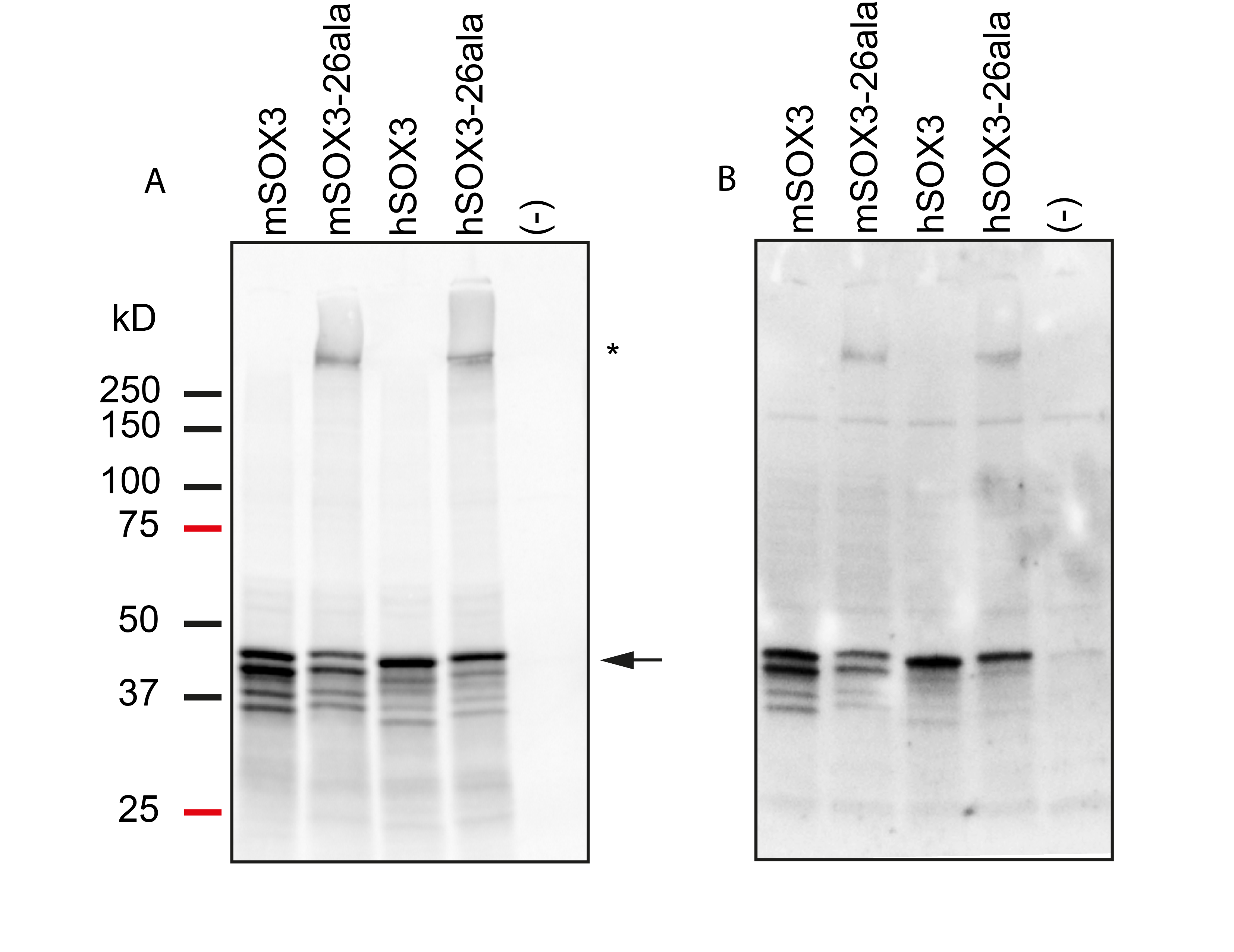

Supplement: Figure S4 — A) Phospor image of an in vitro transcription/translation reaction of mouse and human SOX3 WT and 26ala expression constructs. SOX3 protein of the expected size is indicated by the arrow. Two strong bands were generated by the mouse plasmids, possibly reflecting the inclusion of an upstream ATG in the plasmid, while human plasmids generated only one strong band at the expected size. SOX3-26ala for both mouse and human runs slightly higher than WT protein reflecting the expanded polyalanine tract. In addition, both mouse and human SOX3-26ala have a high molecular weight species that appears to be an aggregated form of SOX3 (*). (-) indicates no template control. B) Immunoblotting using anti-SOX3 antibody (R&D #AF2569) generates a near identical pattern to the phosphor image in (A), thus confirming that the antibody is able to detect mutant and wild type protein with comparable efficiency. (TIF) [file pgen.1003290.s004.tif]
